# Cost consequence analysis of transcutaneous tibial nerve stimulation (TTNS) for urinary incontinence in care home residents alongside a randomised controlled trial

**DOI:** 10.1186/s12877-023-04459-z

**Published:** 2023-11-22

**Authors:** Linda Fenocchi, Helen Mason, Lisa Macaulay, Catriona O’Dolan, Shaun Treweek, Joanne Booth

**Affiliations:** 1 Yunus Centre for Social Business and Health, Glasgow Caledonian University, Glasgow, G4 0BA UK; 2 NMAHP Research Unit, University of Stirling, Stirling, FK9 4LA UK; 3 Health Services Research Unit, University of Aberdeen, Aberdeen, AB25 2ZD UK; 4 School of Health and Life Sciences, Glasgow Caledonian University, Glasgow, G4 0BA UK

**Keywords:** Economic evaluation, Cost consequence analysis, Care homes, Urinary incontinence, Tibial nerve stimulation

## Abstract

**Background:**

Urinary incontinence (UI) is prevalent in more than half of residents of nursing and residential care homes and can have a detrimental impact on dignity and quality of life. Care homes predominantly use absorbent pads to contain UI rather than actively treat the condition. Transcutaneous tibial nerve stimulation (TTNS) is a non-invasive, safe, low-cost intervention with demonstrated effectiveness for reducing UI in adults. We examined the costs and consequences of delivering TTNS to care home residents in comparison to sham (inactive) electrical stimulation.

**Methods:**

A cost consequence analysis approach was used to assemble and present the resource use and outcome data for the ELECTRIC trial which randomised 406 residents with UI from 37 care homes in the United Kingdom to receive 12 sessions of 30 min of either TTNS or sham (inactive) TTNS. TTNS was administered by care home staff over 6 weeks. Health state utility was measured using DEMQOL-U and DEMQOL-PROXY-U at baseline, 6 weeks and 18 weeks follow-up. Staff completed a resource use questionnaire at baseline, 6 weeks and 18 weeks follow-up, which also assessed use of absorbent pads.

**Results:**

HRQoL did not change significantly in either randomised group. Delivery of TTNS was estimated to cost £81.20 per participant, plus training and support costs of £121.03 per staff member. 85% of participants needed toilet assistance as routine, on average requiring one or two staff members to be involved 4 or 5 times in each 24 h. Daily use of mobility aids and other assistive devices to use the toilet were reported. The value of staff time to assist residents to use the toilet (assuming an average of 5 min per resident per visit) was estimated as £19.17 (SD 13.22) for TTNS and £17.30 (SD 13.33) for sham (per resident in a 24-hour period).

**Conclusions:**

Use of TTNS to treat UI in care home residents did not lead to changes in resource use, particularly any reduction in the use of absorbent pads and no cost benefits for TTNS were shown. Managing continence in care homes is labour intensive, requiring both high levels of staff time and use of equipment aids.

**Trial registration:**

ISRCTN98415244, registered 25/04/2018. NCT03248362 (Clinical trial.gov number), registered 14/08//2017.

**Supplementary Information:**

The online version contains supplementary material available at 10.1186/s12877-023-04459-z.

## Background

Urinary incontinence (UI) is a distressing condition for older adults that negatively impacts their dignity and quality of life [1]. With the number of individuals affected by UI predicted to increase rapidly as the population ages, the cost of treating and managing UI is also set to increase [2]. However there is a lack of information about the economic costs of treating UI: a review of 86 trials of anticholinergic drugs prescribed for incontinence noted that no costs or economic measures had been reported in any of the included trials [3]. The highest prevalence of UI is found in older adults living in nursing and residential care homes [4]. More than half of care home residents are estimated to be living with UI [5]. Care homes predominantly use absorbent pads to contain UI rather than actively treat the condition [6]. The NHS currently spends upwards of £80 million a year on absorbent pads alone for the purpose of containing UI [5]. This excludes the costs of associated care needed to manage their use in older frailer individuals or those living with dementia. One potential active intervention for UI is transcutaneous tibial nerve stimulation (TTNS), which uses low frequency electrical stimulation applied to the ankle in a programme of 12 half hour sessions, to stimulate the nerves controlling bladder sensation and reduce symptoms of urgency to void [7]. TTNS is a non-invasive, safe intervention with potential effectiveness for reducing UI in older adults [8]. However, there is little evidence regarding cost-effectiveness of TTNS and although a few studies have reported costs for non-surgical treatments for UI these did not involve TTNS [9–11].

The ELECtric Tibial nerve stimulation to Reduce Incontinence in Care Homes (ELECTRIC) trial was a multicentre, sham stimulation controlled randomised trial to compare effectiveness of TTNS with sham stimulation to reduce volume of UI in care home residents [12, 13]. In addition, a longitudinal, mixed methods process evaluation explored acceptability of the intervention as well as intervention delivery fidelity and support. A published protocol [12] and full trial report [13] are available. Thirty-seven care homes (nursing and residential) in England and Scotland took part. Alongside the trial an economic evaluation compared TTNS to sham (inactive) electrical stimulation. This article reports the results of a Cost Consequence Analysis (CCA) which examined the costs of providing TTNS for frail older residents in care homes, changes in resource use in each trial group over time, and assessed the impact on health-related quality of life (HRQoL). Data about HRQoL was measured using DEMQOL and DEMQOL-PROXY, outcome measures designed specifically for use with individuals experiencing cognitive decline and dementia [14, 15]. DEMQOL-U consists of five domains: positive emotion, memory, relationship, negative emotion, loneliness. There are four possible levels of response relating to severity available. DEMQOL-PROXY-U consists of four domains (as DEMQOL-U but without loneliness domain) and has four response levels. The original intention was to use the DEMQOL and DEMQOL-PROXY for cost-effectiveness analysis (CEA) to complement the CCA. However, as no significant difference in primary outcomes or outcomes used for cost-effectiveness were observed, it was decided that it was inappropriate to continue to produce incremental cost-effectiveness ratios between the two groups. For the economic analysis the assumption was made that the sham stimulation group represented usual continence care (excluding the sham stimulation). Baseline data was used to establish the usual continence care pathways in care homes in terms of resource use patterns. The aim of the analysis was to estimate the costs and consequences of TTNS in care homes, summarising the resource use and outcome data in a CCA balance sheet.

## Methods

### Participants and setting

Participants in this study were from the ELECTRIC trial: 25/04/2018, ISRCTN98415244 and ClinicalTrials.gov NCT03248362 14/08/2017. A CONSORT diagram can be found in the full NHS Health Technology Assessment (HTA) report available online [13], and a copy is provided as a supplementary material (Additional file 1). Participants were older adults residing in care homes, including those with cognitive impairment, who experienced UI at least weekly, used the toilet or a toilet-aid for bladder emptying, with or without assistance, and wore absorbent pads to contain urine. Residents were not eligible if they had an indwelling urinary catheter; symptomatic urinary tract infection; post-void residual urine volume more than 300ml; a cardiac pacemaker; treated epilepsy; bilateral leg ulcers; current pelvic cancer; palliative care status, or were non-English speakers.

### TTNS intervention

Participants received an electrical stimulation programme comprising 12 sessions of 30 minutes’ duration each. Delivered twice weekly over 6 weeks using a portable machine (Neurotrac Continence™), two surface electrodes were applied to the ankle to electrically stimulate the tibial nerve. Delivery of each session required one care home staff member to attach and set up the machine, and remove it once treatment was complete. Continual presence of staff members was not required during the stimulation session. Each staff member received a specific package of training to deliver TTNS, including a handbook and DVD and ongoing support by an Implementation Support Facilitator, if needed.

### Economic evaluation

An economic evaluation of TTNS compared with usual continence care was completed in the form of a CCA [16] and results were presented in a CCA balance sheet. Descriptive presentation of the array of effects of the intervention enables decision makers to form their own opinion on relevance and relative importance of the findings to their decision making context [17]. Outcomes, such as use of incontinence products or impacts on continence care pathways, were reported in natural units. Costs were reported in GBP 2018–2019 prices. Discounting was not required. To provide a practical assessment of resource implications and consequences of interest for those involved in care home service provision and making funding decisions a public sector payer (central government Treasury) perspective was used.

### Data collection

Baseline demographic data was collected in the main trial and included age, gender, UI severity, need for help to use the toilet. Individual resident absorbent pad usage in 24 h (brand, product name, size and number used) was measured at baseline, 6, 12 and 18 weeks using diaries kept by care home staff which were counted by the Research Assistants. The staff grade of the person delivering TTNS or sham stimulation and average time taken to set up and remove equipment (over 6 week period (minutes) was recorded by trained care home staff. Participant HRQoL was measured using DEMQOL and DEMQOL-PROXY (titled DEMQOL-Carer) questionnaires at baseline, 6 and 18 weeks [14, 15]. Raw scores were converted to a single utility index for DEMQOL-U and for DEMQOL-PROXY-U using the scoring algorithm for the UK [18, 19]. A Resource Use Questionnaire (RUQ) designed for this study was used to collect data about resources required for toilet assistance, aids and devices for managing incontinence, medication prescribed for overactive bladder symptoms and incontinence, and use of primary care. The RUQ was administered at baseline to establish the usual continence care pathway and completed by a Research Assistant. Data about appointments with health services staff for continence problems were available at 6 weeks and 18 weeks (each covering the preceding 6 weeks).

### Unit costs

Unit costs were attached to the individual resources identified in the RUQ using Unit Costs of Health and Social Care for staff for primary care, and British National Formulary for prescribed medication [20, 21] (Table 1). Data about consultations with health care professionals external to the care home, specific to UI, was categorised according to staff grade and location. Costs for incontinence products and equipment to deliver the intervention (TTNS Neurotrac™ machine, consumables (skin electrodes, wipes, batteries)) were based on market rates for these items. Direct costs included expenditure on absorbent pads and expenditure on other protection products to manage urinary incontinence. Cost of toileting assistance was based on an assumed average of 5 min per resident per visit, and a derived average hourly pay of £36.08 per staff member (based on unit costs for proportions of staff delivering intervention [source: trial data]).

**Table 1:** Applied unit costs and data sources for unit cost valuation

Cost category	Applied unit cost (£) per recorded item (2019 prices)	Assumption for units used in calculations
TTNS training: time to deliver	143.00	Trial records (estimation £25.00 per hour); based on 2 trainers for 2 h per event, plus additional travel time and mileage reimbursement (£0.45 per mile) per person of 30 min for an average round trip of 20 miles.
TTNS training: materials (Handbook and DVD)	11.20	Trial records (estimation); per pack per trainee.
TTNS delivery: Neurotrac machine (branded)	74.49	Per machine. 172 units were used during ELECTRIC trial.
TTNS delivery: PP3 alkaline battery (Energiser brand)	1.70	Based on assumption of replacement (one battery) every 10 h of use
TTNS delivery: Skin electrode pads (single use)	2.98	Based on two pads per stimulation episode*
TTNS delivery: Staff time per stimulation episode	38.06	Average salary estimated from staff roles reported in trial stimulation diary records (66% care assistants, 13% care leaders, 20% nurses).
TTNS support: individual staff competency assessment	31.50	Trial records (estimation); based on one implementation support facilitator for 1 h per assessment (plus travel cost).
**Incontinence products**		
Adult pants	0.906	Based on average cost (range £0.306 to £1.460) (supplier websites**)
Bed & chair protection	6.860	(supplier websites**)
Pads	0.377	Based on average cost (range £0.105 to £1.425) (supplier websites**)
Slips (all-in-one taped products)	0.728	Based on average cost (range £0.461 to £1.114) (supplier websites**)
Community-based social care staff***		
AfC Band 5 nurse	60.00	Per hour of patient-related work.
Care Assistant	28.00	Based on face-to-face, per hour, weekday
Care Leader	40.00	Per hour.
Community-based health care staff***		
Continence nurse (AfC Band 6)	50.85	Based on assumed duration of 27 min (as per practice nurse home visit), costed as the time of one hospital-based nurse specialist.
District nurse (AfC Band 6)	37.80	Based on assumed duration of 27 min (as per practice nurse home visit) of patient-related work
GP	39.00	Based on 9.22-min consultation
GP care home visit	100.62	Based on assumed duration of 23.4-min consultation [PSSRU Unit Costs 2013, workload survey]
GP Practice nurse	9.25	Based on assumed duration of 15-min consultation
GP Practice nurse care home visit	16.65	Based on 27-min consultation
Occupational therapist	44.00	per hour
Physiotherapist (Community Services)	46.00	per hour
Medications used to treat urinary incontinence (unit cost per tablet (£)****)		
Duloxetine	0.12	20 mg 28 capsule
Fesoterodine fumarate	0.92	4 mg 28 tablet
Mirabegron	0.97	25 mg 30 tablet
Oxybutynin hydrochloride	0.02	2.5 mg 84 tablet
Solifenacin succinate	0.92	5 mg 30 tablet
Tolterodine tartrate	0.52	1 mg 56 tablet
Trospium chloride	0.43	20 mg 60 tablet

AfC, Agenda for Change; GP, General Practitioner; TTNS, transcutaneous posterior tibial nerve stimulation

Data collected in trial and using Resource Use Questionnaire were source for units used in calculations

* Costs based on expectation of single use. It could be possible in normal working practice to reuse pads for three to six sessions

** Incontinence products’ market price was identified from supplier direct websites or large chain shops with an online presence. The following supplier websites were used: ID-DIRECT.COM, incontinencepadsdirect.co.uk, incontinencechoice.co.uk, groceries.asda.com, BOOTS.COM, superdrug.com. [Accessed 20 May 2020]

*** PSSRU [21]: Table 7.1 NHS reference costs for hospital services; Table 10.1 Nurses; Table 10.2 Nurse (GP practice); Table 10.3b General Practitioner; Table 11.4 Community occupational therapist (local authority); Table 11.5 Home care worker; Table 11.6 Home care manager; Table 13. Hospital-based nurses

**** from Indicative Drug tariff price (BNF) (2018-19), Joint Formulary Committee “British National Formulary (online) London: BMJ Group and Pharmaceutical Press < http://www.medicinescomplete.com [Accessed on 27 May 2020].“

### Intervention costs

Training was delivered by trial staff and an allocated cost was estimated which included time for face-to-face delivery and travel to individual care homes. As training would not be expected to be delivered off site in normal working practice the cost of training facilities was not included. The sunk costs for development of the Handbook and DVD were not included. Care home staff time for training and time to deliver the intervention were costed using staff roles reported for delivery of TTNS/sham intervention. Sources which were used to estimate total cost per participant are given in Table 1.

### Data analysis

The total cost per participant was estimated by combining the number of each item of resource used with the unit cost of that item. This provided an estimate of mean cost per participant by treatment group. Differences in mean costs associated with UI products, staff time for toilet assistance and other health care resource use (e.g. GP visits) during routine follow-up were estimated. Independent samples t-test was used to compare resource use by the groups at each time point. Utility change scores were assessed for differences before and after TTNS (6 weeks), at 18 weeks follow up and between groups at each time point.

### Missing data

When no data was present for health care NHS contacts, the participant was assumed not to have used the resource category. Data was not imputed for missing DEMQOL or DEMQOL-PROXY responses.

## Results

### RCT results

The ELECTRIC trial randomised 406 residents with UI from 37 care homes in the United Kingdom (23 Scotland, 14 England). 76% of participants were female, average age 85.5 years (range 58 to 107 years). Trial data indicated that participants (TTNS, *n* = 197; Sham, *n* = 209) were similar between arms, that most had severe UI of 400ml per 24 h or more and all wore absorbent pads to manage their UI. The trial primary outcome was volume of urine leaked into pads and the data indicated that TTNS was not superior to sham stimulation in reducing leakage of urine over 24 h.

### Quality of life outcomes

No significant difference was found between participants’ HRQoL scores over time, or between treatment and control groups at any time point, for either DEMQOL-U or DEMQOL-PROXY-U (Table 2). Baseline utility scores could be calculated using DEMQOL-U for 35% (*n* = 141) participants, and using DEMQOL-Proxy-U for 98% (*n* = 397) participants. Minimum and maximum utility values were reported for both tools at baseline (DEMQOL-U 0.243 to 0.986 and DEMQOL-PROXY-U 0.363 to 0.937). A larger range of mean utility values was observed for DEMQOL-PROXY (0.722 to 0.742) than DEMQOL (0.790 to 0.803) reported by the care home resident. As no evidence of clinical effectiveness was found, and no difference in HRQoL was observed, no further synthesis of costs and benefits to produce incremental cost-effectiveness ratio (ICER) between the two arms was conducted.

**Table 2:** Participant health-related quality of life measures at baseline, 6-weeks and 18-weeks follow-up, by randomised group

Assessment	TTNS (*n* = 197)	Sham (*n* = 209)	Mean difference (95% CI)	*p*-value
	Mean (SD); *n*	Mean (SD); *n*		
**DEMQOL-U**				
Baseline	0.771 (0.187); 66	0.826 (0.162); 75		
6 weeks	0.774 (0.203); 48	0.803 (0.156); 59	-0.029 (-0.098, 0.040)	0.405
18 weeks	0.778 (0.171); 28	0.821 (0.143); 40	-0.042 (-0.119, 0.034)	0.271
**DEMQOL-PROXY-U**				
Baseline	0.716 (0.123); 190	0.727 (0.122); 207		
6 weeks	0.730 (0.122); 159	0.736 (0.122); 165	-0.006 (-0.033, 0.021)	0.653
18 weeks	0.741 (0.117); 129	0.744 (0.128); 150	-0.002 (-0.031, 0.027)	0.877

SD, standard deviation

### Resource use for continence care

There was very little change in staff time to manage UI, use of assistive devices for visiting the toilet, use of incontinence products to manage UI, primary care appointments related to UI or medication for UI during the study. Table 3 indicates that 85% of participants needed toilet assistance as routine, on average requiring one or two staff members to be involved four or five times in each 24 h. No significant difference was found between TTNS and sham for the value of staff time to assist residents to attend the toilet: £19.17 (SD 13.22) for TTNS and £17.30 (SD 13.33) for sham (per resident in a 24 h period). Similar proportions of special equipment (898 items (433 TTNS, 465 sham)) were used on a daily basis across the groups: mobility aid (~ 40%), transfer aid (~ 25%) and toilet aid (~ 20%). No significant difference was found between groups for use of products related to incontinence management: the average cost was £1.19 (SD £1.51) per participant in 24 h. Low use of primary care health care professionals and very few medications specifically prescribed for UI were reported. 53 participants (21 TTNS; 33 sham) used health care services for their UI. Most contacts were GP surgery appointments (*n* = 75) or GP care home visits (*n* = 49), accounting for 79% of all contacts. No significant differences were observed between groups.

**Table 3:** Resource use summary, by randomised group

	Trial arm (timepoint in weeks)							
	TTNS				Sham			
Item	0	6	12	18	0	6	12	18
**Toileting assistance**								
**Participants requiring assistance of staff to visit toilet, n**	169*	**158**	**--**	**131**	178*	**159**	**--**	**139**
Number visits to toilet in 24 h, mean (SD)	4.07 (2.51)	4.68 (1.34)	**--**	4.20 (2.70)	3.88 (2.72)	4.24 (2.71)	**--**	3.73 (2.71)
Staff required per visit to toilet, mean (SD)	1.48 (0.73)	1.34 (0.67)	**--**	1.39 (0.67)	1.30 (0.68)	1.29 (0.73)	**--**	1.26 (0.75)
Estimated cost per resident in 24 h (value of staff time), mean (SD) in GBP (£)	18.86 (12.99)		**--**	18.13 (13.06)	16.38 (12.47)		**--**	16.33 (13.21)
**Participants requiring special equipment to visit toilet, n**	**105**	**96**	**--**	**67**	**117**	**98**	**--**	**86**
…transfer aid items (e.g. hoist, stand aid)	60	39	**--**	29	63	32	**--**	25
…mobility aid items (e.g. walking stick, walking frame)	79	63	**--**	49	86	64	**--**	62
…toileting aid items (e.g. commode, raised toilet seat)	36	31	**--**	18	36	29	**--**	31
**Incontinence management products**								
**Participants using absorbent pads, n**	**197**	**166**	**152**	**133**	**209**	**178**	**156**	**156**
Total pads used in 24 h	769	625	558	476	806	661	552	536
Per participant in 24 h	2.79	2.68	2.44	2.53	2.73	2.51	2.54	2.40
Estimated cost per resident in 24 h, mean (SD) in GBP (£)	1.25 (1.42)	1.19 (1.21)	1.05 (0.90)	1.27 (2.15)	1.30 (1.53)	1.12 (1.01)	1.13 (0.92)	1.22 (2.43)
**Community-based health care services, number of appointments**								
**Participants reporting use of services for continence, n [NC]**	**--**	**13**	**--**	**8**	**--**	**24**	**--**	**15**
General Practitioner	**--**	28	**--**	16	**--**	50	**--**	30
Practice nurse / district nurse	**--**	10	**--**	0	**--**	7	**--**	2
Physiotherapist/occupational therapist	**--**	0	**--**	0	**--**	2	**--**	1
Continence service	**--**	0	**--**	2	**--**	7	**--**	2
Average cost for primary care health services used in preceding 6-weeks, mean (95% CI) in GBP (£)		11.88 (5.72 to 18.77)		7.66 (2.52 to 14.25)		19.40 (11.62 to 27.93)		12.60 (6.81 to 19.24)
**UI medication**								
**Participants reporting UI medication, n [NC]**	**19**	**14**	**--**	**11**	**10**	**8**	**--**	**10**
Duloxetine	2	2	**--**	2	1	1	**--**	1
Fesoterodine fumarate	0	0	**--**	0	1	1	**--**	1
Mirabegron	5	4	**--**	3	2	0	**--**	1
Oxybutynin hydrochloride	5	4	**--**	2	1	1	**--**	2
Solifenacin succinate	4	2	**--**	2	3	3	**--**	3
Tolterodine tartrate	2	2	**--**	2	0	0	**--**	0
Trospium chloride	1	0	**--**	0	2	2	**--**	2

*more than 85% of participants; RUQ data (collected at 0, 6 and 18 weeks) used for toileting assistance and community-based health care services categories. Trial data (collected at 0, 6, 12 and 18 weeks) used for incontinence products categories (absorbent pads, adult pants, all-in-one slips, bed and chair protection)

GBP, pound sterling (currency); NC, not costed; SD, standard deviation; TTNS, Transcutaneous Tibial Nerve Stimulation; UI, urinary incontinence

### Cost of TTNS intervention

The average cost of the training and support package per staff member was estimated to be £121.03 (based on assumption of local trainers (10-mile radius) and excluding economic cost of venue). The cost of delivery of TTNS (exclusive of training) in the trial was estimated to be £81.20 per participant (Table 4).

**Table 4:** Cost of training activities and TTNS intervention

Item	Total cost (£)
**TTNS Training and Support Package**	
425 Training Handbook and DVD	4,760.00
72 training events (cost of trainers)	10,296.00
Attendance at 2-hour intervention training – cover costs	
170 Carers	9,520.00
210 Care Leaders	16,800.00
45 Nurses	5,400.00
148 Individual staff competency assessment	4,662.00
Average training cost per staff (*n* = 425)	121.03
**TTNS intervention**	
172 units (electrical stimulator: Neurotrac Continence™)	12,812.28
Skin electrodes (single use) for 4130 stimulation episodes	12,307.40
206 h (4127 set up/remove activities (per resident per stimulation event), 3 min each	7,440.98
Average intervention cost per participant (*n* = 406)	81.20

Trial data as source for all estimates. NOTE: 4130 stimulation event attempts were reported. Three were excluded due to insufficient data

### Cost effectiveness

The trial indicated there was no evidence of clinical effectiveness of TTNS. In the absence of benefit, a claim for cost-effectiveness cannot be made unless there is a reduction in resource use resulting from the new intervention. The use of TTNS did not reduce resources use in terms of staff time or continence pad usage compared to current practice. As the TTNS intervention is in addition to usual care and therefore incurs an additional cost it is not cost effective.

### CCA balance sheet

A descriptive comparison of the costs and outcomes (clinical and economic) of the ELECTRIC trial is presented in the cost-consequence balance sheet (Table 5).

**Table 5:** Descriptive cost consequence analysis balance sheet for the ELECTRIC trial

In favour of TTNS	In favour of current practice
• In contrast to usual continence care which seeks to contain UI, TTNS is a treatment seeking to attempt to address the cause of UI. It is non-invasive, acceptable to Care Home resident and tolerated well. • Staff time to set up/take off the machine was 5 min or less for 94% of stimulations. • Staff do not require to be present for the duration of treatment.	• ITT complete case analysis favoured the Sham intervention statistically but this difference was not considered to be clinically important (trial results)
**Neither in favour of nor against TTNS**	
• No impact on absorbent product use. Average cost of £1.19 (SD £1.51) per participant in 24 h, during trial. [Mean difference (SD) between TTNS and Sham at 18 weeks: number of pads per day, 0.13 (-0.160, 0.416); value of pads (£), -1.16 (-0.400, 0.492)]. • No evidence of impact on health-related quality of life (as measured using DEMQOL-U/DEMQOL-Proxy-U). [Mean difference (SD) between TTNS and Sham at 18 weeks: DEMQOL-U, -0.042 (-0.119, 0.034); DEMQOL-Proxy-U, -0.002 (-0.031, 0.027)]. • No impact on resources (staff time, equipment) required for residents’ toilet assistance. Dependency on staff for toilet assistance was indicative of labour intensity of continence care. [Estimated value of staff time (£) per 24-hour period, assuming 5 min per visit: TTNS, 19.17 (SD 13.22); Sham 17.30 (SD 13.33)]. • Additional cost per resident to receive TTNS (cost of machine and electrodes) during the trial was estimated as £81.20. This proportional cost would lower with active reuse of electrodes and would change further depending on both the lifetime of the machine and also the number of residents that might be expected to use the machine. Cost of staff training and support during the trial was estimated as £121.03 per staff member.	

## Discussion

The economic analysis indicated that for the care homes involved in the study there was no meaningful change in resource use associated with continence care over time (pads, toileting assistance, health care use). Although costs were slightly lower for residents receiving TTNS than for the sham group differences were not significant. Changes to usual care pathways for continence care during the trial were the delivery of TTNS/sham intervention and the 24-hour pad collection. The latter being a trial specific activity which would not need to be continued in normal working practice. In line with the main findings of the ELECTRIC trial which reported that TTNS was not found to be clinically effective in this population [22], no evidence of impact on HRQoL (as measured using DEMQOL-U/DEMQOL-Proxy-U) was observed. In addition to the challenges of blinding, poor information about resident’s type of UI and the degree of physical and/or cognitive frailty of participants (see Trial report for discussion of limitations [22]), the developers of the DEMQOL utility index have highlighted that the health state classification system may be limited for individuals with severe dementia [18]. The mean participant Mini Mental State Examination score was 13.1 (SD 9.1) indicating the resident sample had predominantly moderate to severe dementia. A further challenge is that DEMQOL-U and DEMQOL-PROXY-U have been suggested to be more responsive to changes in dementia symptoms than to physical changes [23]. While data about quality of life was successfully collected using both DEMQOL-U and DEMQOL-PROXY-U, completion of DEMQOL by participants was fewer, reflecting the severity of their cognitive impairment. It should also be noted that as the proxy form could be completed by a different person at each of the three data collection timepoints, consistency of response cannot be assumed.

It was notable from the resource data that participants’ dependency on staff to take them to the toilet and the use of transfer and mobility aids were a daily and ongoing requirement. This was reflected in the observed lack of changes in continence care practices and therefore costs of the existing continence care pathways in care homes. If TTNS is effective then residents would be expected to experience a reduced sensation of urinary urgency and increased warning time for the need to void. However, if no additional toilet visits were provided to enable residents to void in a toilet, as opposed to using the absorbent pads to void, any effects produced by TTNS would be unlikely to be recognised. Incontinence in care homes is usually managed using absorbent pads. These can be costly to the care home and health service providers. Usage of continence products to manage UI did not change between timepoints and although costs were slightly lower for residents receiving TTNS than sham, differences were not significant. Only a small number of individuals reported use of primary care health professionals for incontinence related issues. Similarly, a small number of participants were reported as receiving medications prescribed for UI. The absence of anticholinergic medications and the very low reported use of the NHS continence service during the trial period confirmed that the usual continence care approach was to seek to contain UI rather than instigate active treatment for UI, in line with other studies [6, 24].

A cost consequence analysis approach was useful to indicate which costs and outcomes will be most relevant to future continence trials in care homes. Qualitative evidence indicated that a positive impact on laundry (a reduction in items requiring laundering due to UI) had been noted by one care home manager [22]. Data had not been collected about this during the trial and future studies may wish to consider including the collection of such resource data. For the economic analysis, retail prices were used to cost continence products. In practice, lower unit costs per product could be attainable by care homes if they have supplier agreements in place. The main trial results reported no treatment difference between the groups, including for subgroup analysis by falls status [22]. For the economic component of the study a pragmatic decision was made to not collect data on secondary care beyond use of services for continence in order to minimise the amount of data care home staff were required to report. This was based on the view that TTNS was not expected to change any care given to people other than potentially affecting clinical degree of incontinence and thereafter resultant care home continence care pathway practices. However, UI is a known risk factor for falls in care home adults and consequent increased health care costs and so is an important outcome that should be explicitly considered for data collection on a study by study basis. It is the case that staff time is a significant portion of continence-related costs in care homes. The high dependency of residents (physical and cognitive) in general, and high proportion of residents requiring assistance to use a toilet, often compounded by severity of UI, presents practical constraints to continence care in this setting. To impact this in a substantial way is challenging. Research to explore the effects of in-depth training about UI for care home staff, to improve staff knowledge and understanding about causes and types of incontinence, effects of incontinence on residents and different management strategies could provide useful insight.

### Implications

Our study adds economic evidence of the costs and consequences of delivering TTNS using electrical stimulation to reduce UI in older care home residents. It suggests TTNS does not impact on current resource use for the management of UI in care homes. However, the trial also found no evidence of clinical effectiveness and no effect on HRQoL of TTNS for older care home residents.

## Conclusions

In summary, the evidence from the CCA of the ELECTRIC trial does not suggest that there is an economic case for TTNS in the care home context. The use of TTNS does not change the volume or type of resources used to manage continence in care homes. Residents in both randomised groups continued to receive high levels of staff assistance to use the toilet, use of aids and devices for managing incontinence, including absorbent pads, without significant difference from baseline.

### Electronic supplementary material

Below is the link to the electronic supplementary material.

Additional file 1: A CONSORT flow diagram of care homes and participants through the ELECTRIC Trial.

## Data Availability

The datasets used and/or analysed during the current study available from the corresponding author on reasonable request.

## List of Abbreviations

CCA: Cost consequence analysis
CEA: Cost effectiveness analysis
CPD: Continuing professional development
DEMQOL: Measure of health-related quality of life for people with dementia
DEMQOL-U: Preference-based single index for DEMQOL
DEMQOL-PROXY-U: Preference-based single index for DEMQOL-PROXY
ELECTRIC trial: ELECtric Tibial nerve stimulation to Reduce Incontinence in Care Homes Trial
HRQoL: Health-related quality of life
TTNS: Transcutaneous posterior tibial nerve stimulation
UI: Urinary incontinence
