# Supplementary material for: Cost consequence analysis of transcutaneous tibial nerve stimulation (TTNS) for urinary incontinence in care home residents alongside a randomised controlled trial
Source: BMC Geriatr. 2023 Nov 22;23:766. doi: 10.1186/s12877-023-04459-z (PMC10666345; doi:10.1186/s12877-023-04459-z)
Supplement: Supplementary file 1 — Additional file 1: A CONSORT flow diagram of care homes and participants through the ELECTRIC Trial. [file 12877_2023_4459_MOESM1_ESM.docx]

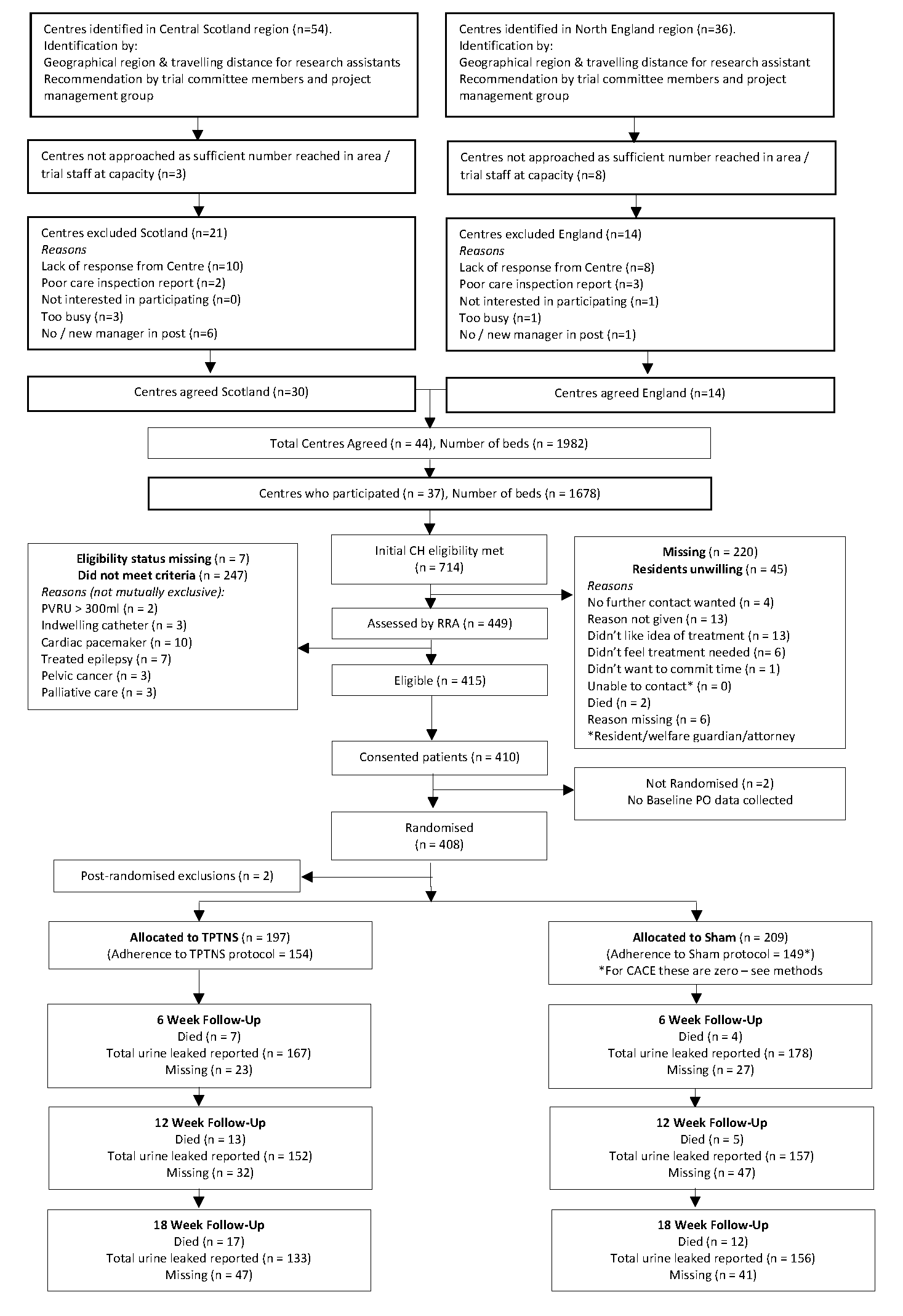


**CONSORT flow diagram of care homes and participants through the ELECTRIC Trial**

CH = care home; PVRU = Post void residual urine; RRA= Regional Research Assistant; TPTNS = Transcutaneous posterior tibial nerve stimulation; REL = Resident Eligibility Log
